# Enhancing MALDI Time-Of-Flight Mass Spectrometer Performance through Spectrum Averaging

**DOI:** 10.1371/journal.pone.0120932

**Published:** 2015-03-23

**Authors:** Morgan Mitchell, Sujina Mali, Charles C. King, Steven J. Bark

**Affiliations:** 1 Department of Biology and Biochemistry, University of Houston, Houston, Texas, United States of America; 2 Department of Pediatrics, Pediatric Diabetes Research Center, The University of California San Diego, San Diego, California, United States of America; University of Parma, ITALY

## Abstract

Matrix-assisted laser desorption ionization time-of-flight (MALDI-TOF) mass spectrometers are simple and robust mass spectrometers used for analysis of biologically relevant molecules in diverse fields including pathogen identification, imaging mass spectrometry, and natural products chemistry. Despite high nominal resolution and accuracy, we have observed significant variability where 30–50% of individual replicate measurements have errors in excess of 5 parts-per-million, even when using 5-point internal calibration. Increasing the number of laser shots for each spectrum did not resolve this observed variability. What is responsible for our observed variation? Using a modern MALDI-TOF/TOF instrument, we evaluated contributions to variability. Our data suggest a major component of variability is binning of the raw flight time data by the electronics and clock speed of the analog-to-digital (AD) detection system, which requires interpolation by automated peak fitting algorithms and impacts both calibration and the observed mass spectrum. Importantly, the variation observed is predominantly normal in distribution, which implies multiple components contribute to the observed variation and suggests a method to mitigate this variability through spectrum averaging. Restarting the acquisition impacts each spectrum within the electronic error of the AD detector system and defines a new calibration function. Therefore, averaging multiple independent spectra and not a larger number of laser shots leverages this inherent binning error to mitigate variability in accurate MALDI-TOF mass measurements.

## Introduction

Matrix assisted laser desorption time-of-flight (MALDI-TOF) mass spectrometers are highly robust and capable instruments for biomolecular analysis. While the first practical TOF mass spectrometer was developed in the 1950s[1], it was the advent of new ionization techniques compatible with large biological molecules like MALDI[2] and electrospray ionization[3] that revolutionized the biological applications of mass spectrometers including TOF instruments. The rapid advancement of biological mass spectrometry is largely attributable to these ionization techniques and their impact on vastly improved mass spectrometer instrumentation performance. The importance of MALDI-TOF mass spectrometry can be appreciated through brief literature review for diverse fields including identification of bacterial and viral pathogens [4–5], clinical pathology [6–9] imaging mass spectrometry,[10–11] biochemistry and natural products[12–14].

Modern MALDI-TOF mass spectrometers use delayed extraction and ion reflector systems to enhance instrument resolution and accuracy, enabling accurate mass measurements of peptides and molecules (S1 Fig.)[15–16]. Routine performance specifications for reflector MALDI-TOF instruments often exceed 15,000 for resolution measured by full-width at half-maximum (FWHM) and <5 parts-per-million (ppm) for accuracy with internal calibration. This performance is sufficient for most biological applications including protein identification by peptide mass fingerprinting, a technique that is highly dependent on high accuracy mass measurements of component peptides[17]

The MALDI-TOF/TOF mass spectrometer used for these studies is a high performance reflectron instrument with specifications at the level described in the previous paragraph in TOF mode. However, we have observed significant variability in replicate mass measurements from under 1 to 20 ppm or greater on this instrument, even in internally calibrated spectra (S1 and S2 Tables). For example, considering replicate measurements for multiple different peptides, 30–50% of individual measurements exhibited errors in excess of 5 ppm. We have made similar observations for multiple MALDI-TOF-type instruments from different manufacturers, which suggest these factors are intrinsic to this mass spectrometer design. Additionally, mass measurements for multiple different peptides within a single mass spectrum often exhibit uncorrelated errors. Increasing the number of laser shots for each spectrum did not resolve this variability. Unfortunately, there is no *a priori* method to define the accuracy of an unknown peptide mass measurement and, therefore, these observed mass deviations cannot be compensated or mitigated. We hypothesized that understanding the basis for the observed variability in replicate mass measurements could suggest a method to mitigate these errors and improve the consistency of MALDI-TOF measurements. To this end, trypsin digests of both a standard protein mixture and proteins derived from a biological immunoprecipitation experiment were analyzed using a high resolution MALDI-TOF/TOF mass spectrometer in TOF mode with 5-point internal calibration. The same sample was also analyzed using a quadrupole-time-of-flight (Q-TOF) mass spectrometer coupled to a HPLC system. Direct comparison of these different mass spectrometry platforms enhanced identification of peptides and provided high confidence for evaluating accuracy and performance of the MALDI-TOF mass spectrometer.

These data demonstrate significant variability in observed peptide masses and the discontinuous nature of the analog-to-digital (AD) detector system in the MALDI-TOF mass spectrometer. When restarting acquisition, the AD detector system resets the position of the bins within the electronic error of the system, thus shifting the data by a small amount (usually less than the width of a single bin). This error impacts both flight time measurement and calibration function, both of which require interpolation from the discontinuous data observed in the mass spectrum. The data suggest this small error is still significant and contributes to the observed variability in the MALDI-TOF data.

While the mechanisms underlying the variability observed in the MALDI-TOF data appear complex, the data indicate the method to resolve this variability is simple. The bin repositioning for each independent spectrum and calibration follow a normal Gaussian distribution. Therefore, mass spectral measurements can be analyzed by averaging populations of individual spectra and using simple descriptive statistics appropriate for normally distributed data. This simplified statistical approach provides enhanced instrument performance more consistent with the accuracy specifications of a high resolution MALDI-TOF mass spectrometer. An unexpected advantage to this approach was that statistics identified mass measurements exhibiting high standard deviations, suggesting peptide measurements with high potential for error. Finally, we demonstrate this method enhances identification of proteins in trypsin digestion of standard protein samples and a biological sample from an immunoprecipitation experiment. We anticipate that simplified averaging and calibration algorithms including spectrum averaging and descriptive statistical measures can be readily incorporated into automated acquisition software, providing enhanced performance for any MALDI-TOF mass spectrometer.

## Materials and Methods

### Isolation and Culture of Human Embryological Cells

HEK293 cells were purchased from ATCC and grown at 37°C in DMEM/High Glucose media with 10% fetal bovine serum and 1% Penicillin-Streptomycin. At 90% confluence, cells were trypsinized, washed three times with warm deionized phosphate buffered saline, then lysed in buffer containing 1% sodium deoxycholate, 1X protease inhibitor cocktail, and 1% nuclease. Total protein concentration was estimated using 280nm absorbance. All reagents were purchased from Thermo Scientific except for Penicillin-Streptomycin, which was purchased from Gemini BioProducts.

### Cell Lysate Preclearing

Cell lysate (1.275 mg) was centrifuged at 16,000 x g for 40 min at 4°C and the supernatant was added to phosphate buffered saline (PBS) containing 0.1% Tween-20 (0.1% PBST) and allowed to bind to 50 μl of Protein A/G agarose beads (Santa Cruz) for 2 h at 4°C with mixing. After incubation with resin, the supernatant was isolated by centrifugation and used for subsequent experiments immediately or stored at −80°C.

### Preparation of Antibody-Linked Dynabeads

3 mg of Protein G Dynabeads (Life Technologies) were washed three times with 200 μl of PBST. After washing, 24 μg anti-β-tubulin rabbit polyclonal antibody (Abcam) was diluted in 0.1% PBST (200 μl) and incubated with the washed Dynabeads at 37°C for one hour with shaking at 1400rpm. Beads were washed three times with 200 μl of PBST, then conjugation buffer (20 mM Sodium Phosphate, 0.15 M NaCl, pH = 7–9) for crosslinking. Antibody was crosslinked to the Dynabeads using 250 μl of a freshly prepared solution of 5mM Bis (Sulfosuccinimidyl) substrate (BS^3^, Thermo Scientific) and incubated at room temperature for 30 min with shaking at 1400 rpm. The reaction was quenched by adding 1 M Tris HCl, pH = 7.5 (12.5 μl) for 15 min at room temperature. Beads were subsequently washed three times with 200 μl of 0.1% PBST.

### Immunoprecipitation

Anti-tubulin antibody-coupled Dynabeads were resuspended in precleared cell lysate and incubated with rotation at 4°C overnight. After 18 hours, supernatant was decanted and saved for analysis. Beads were washed with 0.1% PBST (200 μl), then 500 mM NaCl (3 x 200 μl), PBS (200 μl) and H_2_O (200 μl). Proteins were eluted using two washes in 20 μl of 5% NH_4_OH for 5 min each wash. Elution fractions were combined and neutralized to pH = 7 by addition of 40 μl of 1M NH_4_HCO_3_ and concentrated *in vacuo*.

### Proteolytic Digests

#### Immunoprecipitated Proteins

Immunoprecipitated tubulin was dissolved in 10 μl of 50% acetonitrile in 100 mM ammonium bicarbonate, pH = 7.2 and reduced and alkylated with TCEP and iodoacetamide. Proteins were precipitated using chloroform/methanol to eliminate residual reagents, detergents, lipids and salts[18]. The protein was then dissolved in 100 mM NH_4_HCO_3_ (60 μl) and 10 μl of a 25 ng/μl trypsin stock solution in 100mM NH_4_HCO_3_ was added. After 18 h incubation at 37°C, 10 μl of 10% formic acid was added to quench the reaction.

#### Purified Proteins

A mixture of BSA, Myoglobin and β-Casein at 1 mg/ml for each protein was dissolved in 100mM ammonium bicarbonate with 1% sodium deoxycholate. Fifty microliters of this standard protein solution was reduced and alkylated with TCEP and iodoacetamide. The deoxycholate concentration was reduced to 0.5% by adding 50 μl of 100 mM ammonium bicarbonate and trypsin was added to concentration of 1/50-1/100 by weight of the protein sample. The digestion was incubated at 37°C for 18 hours. After incubation, the reaction was quenched with 10 μl of 10% formic acid.

### Western Blot Analysis

Immunoprecipitated tubulin proteins were separated by standard gel electrophoresis on Novex 4–12% precast gels and transferred to PVDF membrane using the Novex MiniCell blot module (Life Technologies). Blocking agent was 5% dry nonfat milk in tris-buffered saline (TBS) with 0.1% Tween 20 (TBST). Primary anti-β-tubulin antibody (Abcam) was used at 1:3000 dilution in a 5% solution of bovine serum albumin dissolved in 0.05% TBST. Secondary antibody was horse anti-mouse IgG horseradish peroxidase linked antibody (Cell Signaling) at 1:1500 dilution in a 5% solution of non-fat milk powder dissolved in 0.05% TBST for one hour with gentle shaking. The membrane was developed using the SuperSignal West Pico Chemiluminescent Substrate (Thermo Scientific) according to the manufacturer’s instructions.

### MALDI Time-of-Flight Mass Spectrometry

MALDI-TOF mass spectrometry was performed on an AB Sciex 4800 MALDI-TOF/TOF mass spectrometer using AB Sciex 4000 Series Data Explorer control and processing software (V3.7.1 Build 1, AB Sciex). Protein samples were diluted to approximately 1 pmol/μl, then 0.5 μl of sample was combined with 0.1 μl of a 5-peptide calibration mixture (described below) and 0.5 μl of a saturated solution of α-cyano-4-hydroxycinnamic acid (Sigma Aldrich) dissolved in 50% acetonitrile in water with 1% formic acid and spotted onto a 384-well AB Sciex MALDI plate. Appropriate levels for accurate calibration were determined empirically. Each replicate spectrum acquired was a composite of 500 laser shots followed by 5-Point non-linear calibration using the Data Explorer internal calibration procedure. Calibration employed a mixture of 5 standard peptides at approximately 100 ng peptides per 10 μl of α-cyano-4-hydroxycinnamic acid solution: Des-Arg(9) Bradykinin (MH+ = 904.4676), Human Angiotensin 1 (MH+ = 1296.6848), Glu-1-Fibrinopeptide B (MH+ = 1570.6768), ACTH 1–17 (MH+ = 2093.0862), ACTH 18–39 (MH+ = 2465.1983). All peptides were purchased from Bachem. These peptides were used for plate calibration of the AB Sciex 4800 mass spectrometer prior to analysis followed by close external and internal calibration of all spectra. Close external calibration was acquired by spotting the calibration peptide mixture in close proximity to the standard protein digest sample on plate. Internal calibration incorporated the calibration peptide mixture directly into the protein digest sample on plate. Internal calibration provided more accurate data, but close external calibration provided increased mass spectral signal, presumably from less charge competition between standard and sample peptides. Calibration required 3 bins across peak-width-at-half-height for bin sizes of 0.5 and 1.0 nanosecond and 1 bin for 2.0 nanosecond bin size. No calibration was achieved for 4.0 nanosecond bin size. Bin size modification to 0.5, 1.0, 2.0 and 4.0 nanosecond scale utilized the Digitizer Bin Menu in the Digitizer section of the AB Sciex 4000 Series Data Explorer mass spectrometer operating software. All spectra were required to pass internal calibration to be considered in subsequent analysis. For all experiments, 23 individual spectra were used for analysis to provide enough individual measurements for reliable statistics. However, not all peptides were observed in each spectrum (not uncommon in MALDI-TOF mass spectrometers). Peptide signals exhibiting signal-to-noise lower than approximately 5/1 were not included in reported measurements because of the difficulty in reliable peak assignment. Therefore, the number of individual spectral measurements for each peptide was between 10 and 23 different measurements. To evaluate the contribution of the calibration procedure on the observed variability, the initial calibration function derived from Data Explorer was deleted for multiple spectra and the spectra were subjected to the same calibration procedure as though the spectrum was acquired *de novo*. Multiple recalibrations of the same data provided the exact same calibration functions demonstrating that the calibration algorithm itself is not contributing to the observed data variability.

### Liquid Chromatography Tandem Mass Spectrometry

Liquid chromatography tandem mass spectrometry was performed on a high resolution Bruker MicrOTOF-Q (Q-TOF) mass spectrometer equipped with an Agilent 1200 Nano HPLC system using Solvent A = water with 0.25% formic acid and Solvent B = acetonitrile with 0.25% formic acid. Gradient: 5% Solvent B to 30% Solvent B over 85 min at column flow rate of 1.4 μl/min on a home-packed 75 μm x 100 mm nanobore C18 column. Bruker CaptiveSpray source voltage = 1400 Volts, dry gas = 3.0 L/min, and capillary temperature = 150°C. Data was collected in data-dependent mode fragmentation (MS/MS) on the 3 most intense spectra identified in MS mode. Spectra from the HPLC-MS/MS run were output into an MGF format for analysis in X!Tandem (Current GPM 2013.09.07)[19]. Settings for database search were high resolution Q-TOF (MS tolerance = 100 ppm), fragment mass tolerance = 100 ppm, and trypsin digestion with semi-cleavage allowed, complete acetamidomethylation of cysteine, and potential modifications for methionine and tryptophan (oxidation) and asparagine and glutamine (deamination). Injection volume for BSA sample was 0.05 μl (50 fmole) and 8 μl of tubulin protein sample.

### Descriptive Statistical Analysis

Descriptive statistics were calculated using the statistics functions in Excel (primarily AVERAGE and STDEV) for averaging, standard deviation and descriptive statistical calculation data (Excel 2010, Microsoft Corporation). More advanced statistical calculations such as Shapiro-Wilk normality, p-Value and Grubbs Outlier tests used the SciStat Calc online statistics resource and online documentation of the statistical model algorithms (scistatcalc.blogspot.co.uk). The specific Shapiro-Wilk normality calculation algorithm has been previously adapted from Royston, et al. (Appl. Statist. (1982) Vol. 31, No. 2) by A. Trujillo-Ortiz, R. Hernandez-Walls, K. Barba-Rojo, and L. Cupul-Magana and distributed as a MatLab m-file in Matlab File Exchange.

## Results and Discussion

### Replicate mass measurements and statistical analysis improves consistency of MALDI-TOF data

**Fig 1:**
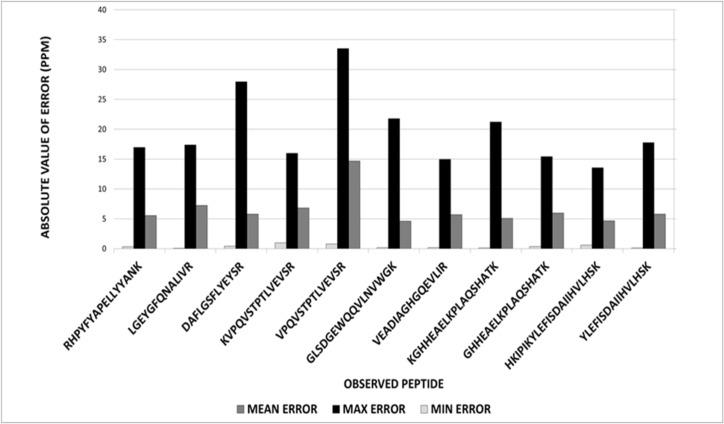
Absolute values for mean, maximum and minimum errors observed for peptides from a standard protein trypsin digestion demonstrate higher variability in the more inaccurate data. Measured errors were converted to absolute values to evaluate the dispersion of data measurements and plotted according to peptide. The uneven distribution of maximum and minimum errors is expected because of zero as a lower bound for minimum error. Note, absolute value transformation of the data eliminates negative values and the mean errors reported here are higher than the mean reported for the observed peptide masses, which contain both positive and negative errors.

**Fig 2:**
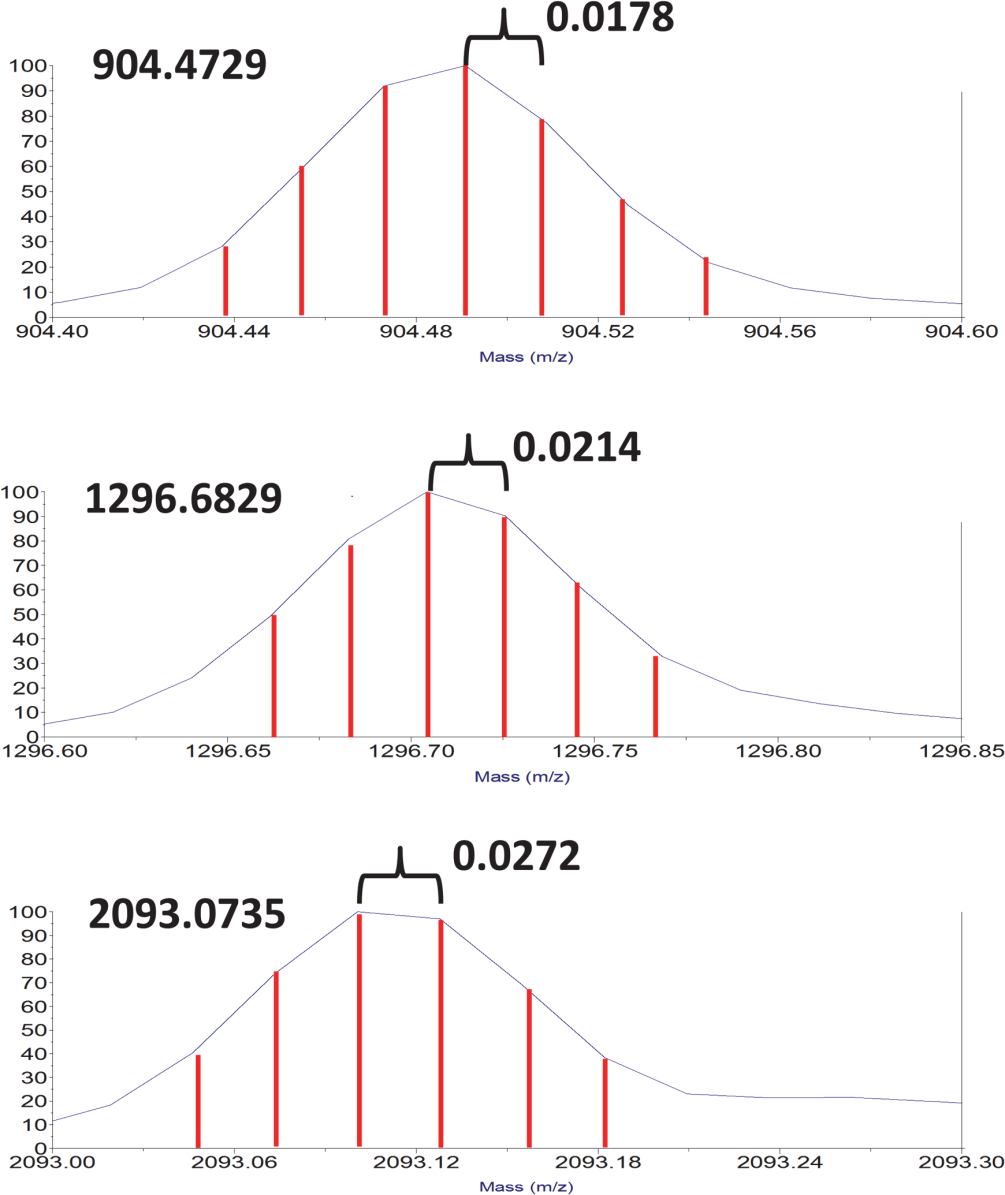
Evidence for discontinuous binning of MALDI-TOF mass spectrometry data for Des-Arg(9) Bradykinin (A), Angiotensin 1 (B), and ACTH 1–17 (C). Representative monoisotopic peaks for Des-Arg(9) Bradykinin (MH+ = 904.4676), Angiotensin 1 (MH+ = 1296.6848) and ACTH 1–17 (MH+ = 2093.0862) are enlarged to demonstrate the discontinuous sampling points (or bins) within the MALDI-TOF data. The red vertical lines are fitted to the bins evident in the observed peak shapes. The spacing of bins in mass units is larger for higher mass ions and can be accurately calculated by relationship between flight times and the ratio of masses of the molecular ions according to the equation Δt_2_/Δt_1_ = (M_2_/M_1_)^1/2^. This calculation is simplistic, but accurately relates the bin spacing for different mass ions to 5 decimal places, thus suggesting that binning of data in the MALDI-TOF instrument is related to the measurements of ion flight times.

To evaluate the underlying mechanism impacting the observed variability in our particular instrument, we analyzed a digest of standard proteins and compiled mass measurements for 11 different identified peptides from 23 individual spectra. Not all peptides were observed in each spectrum and the number of spectral measurements used for analysis of each peptide is indicated in Table 1, Column N. These measurements were subjected to descriptive statistics calculations including mean, standard deviation, Shapiro-Wilk’s normality test, and Grubbs outlier tests (Table 1). In addition to MALDI-TOF analysis, we also subjected these same digests to analysis on a high-resolution Q-TOF LC-MS/MS system for comparison and confirmation of mass measurements. For most peptides, the combined replicate MALDI-TOF mass measurements exhibited a normal distribution as evidenced by Shapiro-Wilk’s normality (Table 1). This observation suggests that several component error factors occur in combination and are essentially random and independent of each other. An immediate observation from these calculations was the improved consistency of the mean peptide mass compared to many of the measured masses from individual spectra. This consistency was observed even in the cases where Shapiro-Wilk’s normality was not confirmed. Absolute values of measured errors evaluate the dispersion of the data and indicate higher variability in the more inaccurate data (Fig. 1) with the standard deviations for the data consistently in the 0.010–0.014 amu range. The peptide mass measurement with the highest standard deviation (0.022 amu) was also the measurement most in error (8.996 ppm, Table 1) and had the greatest dispersion in absolute values of the errors (Fig. 2). We note that a high standard deviation and high dispersion of data may still provide an accurate average, but was not observed in these data. Therefore, these data suggest statistical analysis on a population of individual mass spectral measurements provides more consistently accurate MALDI-TOF data and that data with higher standard deviations may identify mass measurements with higher probability of error.

**Table 1:** Data and statistical calculations for protein standard and tubulin IP peptides.

	Peptide sequence	Protein	Calculated	Q-TOF	MALDI (Mean)	MALDI (SD)	Error (PPM)	N	Ave. S/N	Shapiro-Wilk (PASS)	Outlier
	**Standard Peptides**										
1	RHPYFYAPELLYYANK	BSA	2045.028	2045.028	2045.0191	0.013	−4.36	16	822.19	0.9277(YES)	NO
2	LGEYGFQNALIVR	BSA	1479.795	1479.795	1479.7943	0.01299	−3.62	16	454.81	0.9480(YES)	NO
3	DAFLGSFLYEYSR	BSA	1567.743	1567.743	1567.7391	0.014	−2.29	16	2039.38	0.8519(NO)	NO
4	KVPQVSTPTLVEVSR	BSA	1639.938	1639.938	1639.9326	0.013	2.29	15	381.13	0.9279(YES)	NO
5	VPQVSTPTLVEVSR	BSA	1511.843	1511.8428	1511.8292	0.0223	9.00	16	23.13	0.9612(YES)	NO
6	GLSDGEWQQVLNVWGK	Myoglobin	1815.902	1815.902	1815.8964	0.0119	−3.30	16	391.13	0.8488(NO)	NO
7	VEADIAGHGQEVLIR	Myoglobin	1606.855	1606.855	1606.8511	0.0111	2.25	23	1991.96	0.9738(YES)	NO
8	KGHHEAELKPLAQSHATK	Myoglobin	1982.057	1982.057	1982.0527	0.0138	0.89	19	1870.95	0.9102(YES)	NO
9	GHHEAELKPLAQSHATK	Myoglobin	1853.962	1853.926	1853.9566	0.0121	2.58	20	154.85	0.9379(YES)	NO
10	HKIPIKYLEFISDAIIHVLHSK	Myoglobin	2601.492	2601.492	2601.4832	0.0131	−3.20	12	229.17	0.9707(YES)	NO
11	YLEFISDAIIHVLHSK	Myoglobin	1885.022	1885.022	1885.0142	0.0126	−4.04	15	403.20	0.9476(YES)	NO
	**Tubulin IP Peptides**										
12	LHFFMPGFAPLTSR	Tubulin	1620.836	1620.836	1620.8317	0.0076	−2.37	20	271.50	0.9284(YES)	NO
13	NSSYFVEWIPNNVK	Tubulin	1697.817	1697.817	1697.8129	0.0136	−2.56	18	87.00	0.9723(YES)	NO
14	FPGQLNADLR	Tubulin	1130.595	1130.595	1130.5886	0.0078	−5.92	20	43.35	0.9679(YES)	NO
15	YMACCLLYR	Tubulin	1249.553	1249.553	1249.5846	0.0168	25.57	18	136.67	0.9179(YES)	NO
16	LAVNMVPFPR	Tubulin	1143.634	1143.634	1143.6322	0.0134	−1.87	20	28.35	0.7533(NO)	NO
17	EVDEQMLNVQNK	Tubulin	1446.689	1446.689	1446.7432	0.0159	37.23	17	45.29	0.8645(NO)	NO
18	SYELPDGQVITIGNER	Actin	1790.892	1790.892	1790.8941	0.014	1.25	20	94.25	0.9478(YES)	NO
19	IWHHTFYNELR	Actin	1515.749	1515.749	1515.7469	0.0064	−1.53	18	95.28	0.8983(YES)	NO
20	AMGIMNSFVNDIFER	Hist2H2BF	1743.819	1743.819	1743.824	0.0091	2.67	20	151.85	0.9841(YES)	NO
21	LDIDSPPITAR	PKM2	1197.647	1197.647	1197.6545	0.0176	5.90	17	19.18	0.9180(YES)	NO
22	LISWYDNEFGYSNR	GAPDH	1763.802	1763.802	1763.8219	0.0159	11.04	20	52.00	0.9447(YES)	NO
23	THNLEPYFESFINNLR	Keratin	1993.977	1993.977	1993.9718	0.0002	−2.65	17	109.18	0.9548 (YES)	NO
24*	THNLEPYFESFINNLR	Keratin	1993.977	1993.977	1993.8725	0.298	−52.27	19	98.95	0.3951(NO)	YES

Peptides identified in both MALDI-TOF and LC-MS/MS experiments were subjected multiple replicate measurements on the MALDI-TOF instrument and descriptive statistics were calculated on the population of data for each peptide (12–23 individual measurements from 23 complete spectra, see Materials and Methods). The exact mass for each peptide was calculated in Protein Prospector (UCSF and compared to the masses observed for the Q-TOF LC-MS/MS system (Q-TOF) and the mean of replicate measurements for the MALDI-TOF instrument (MALDI MEAN). The standard deviation (MALDI SD) is reported in amu and the error (Error) was calculated in PPM. The number of replicate measurements (N), the raw Shapiro-Wilk threshold criteria (Shapiro-Wilk (PASS)) and Grubbs Outlier results are reported in their respective columns. Note that Peptide THNLEPYFESFINNLR is listed in line 23 and 24 with all mass spectral measurements (N = 19) for this peptide used for the statistical data in line 24 (Denoted with a *). However, Grubbs Outlier Test analysis indicated positive outliers for two spectral measurements in these data, which were removed (N = 17) to provide the statistical information presented in line 23.

### MALDI-TOF data are comprised of discontinuous bins.

**Fig 3:**
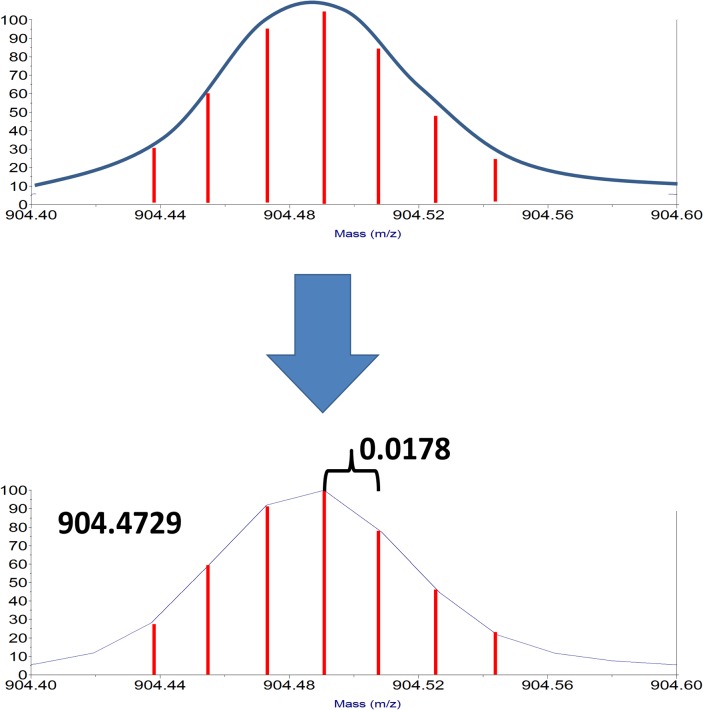
Effect of binning and interpolation in MALDI-TOF data. The signal detected for the flight times of a packet of ions should exhibit a continuum distribution related to the energy distribution of the population of ions (TOP). However, the AD detection system can only measure ion signal from the detector in discrete time intervals represented by the red vertical lines (TOP). The discrete intensity versus time measurements are effectively bins and produce the observed discontinuous peak profile (BOTTOM). This discontinuous data must be interpolated by peak fitting algorithms to estimate the parameters of the original continuum spectrum and derive accurate mass measurements.

Specific evaluation of peak shape in all observed spectra demonstrated discontinuous intensity measurements or sampling points across every peak, suggesting the data was parsed into bins. In the particular mass spectrometer employed for these studies, we observed the discontinuous spacing or bins at 0.0178 amu for Des-Arg(9) Bradykinin (MH+ = 904.4676), 0.0214 amu for Human Angiotensin 1 (MH+ = 1296.6848), and 0.0272 amu for ACTH 1–17 (MH+ = 2093.0862) (Fig. 3). The differences in bin spacing are correlated with the difference in flight times for ions of different masses by a simple ratio equation that accurately relates the bin spacing to 5 decimal places, suggesting strongly that the differences in the ion flight times in the instrument and the binning of data are related (Fig. 2). We note that this treatment is intellectually useful, but an oversimplification. The actual equations for flight times incorporating source and focusing effects in a TOF instrument are considerably more complex than treated here. The interested reader is referred to the excellent article summarizing history and the mathematics of these instruments[20].

The actual signal from flight times for ions in the mass spectrometer should produce a continuum peak shape (Fig. 3). However, the AD detector system can only measure the intensity of ions in discrete packets of signals across the time spectrum defined by the high speed internal clock (500–1000 MHz or higher) and internal electronics (Fig. 3). The data bins widths observed in Fig. 2 correspond to 0.0178 amu for a mass of 904.4729 in the mass spectrometer and increasing for higher masses (0.0214 amu at 1296.6829 and 0.0272 at 2093.0735. These bin widths correlate to 19.7 ppm, 16.5 ppm and 13.0 ppm, respectively. The resulting bin spacing is reasonably consistent with the standard deviation for the peptide measurements (Table 1). These observations are also consistent with our observation that the number of laser shots improve signal-to-noise of the data with little impact on accuracy.

### Multiple mechanisms may impact variability in MALDI-TOF data consistency

The only way to measure the mass of ions in a TOF mass spectrometer is to detect the flight time of the ions from the time of laser shot through delayed extraction to impact at the detector. Our data demonstrate a shifting of bin positions between replicate measurements, which we initially interpreted as variation in the flight time measurements. With each laser shot, the start of acquisition defines the time range and sets the position of the bins. Each subsequent laser shot is an independent measurement and sets new bin positions within the inherent error of the electronics. However, this variability is quite small and usually limited to less than the width of a single bin. For this reason, most MALDI-TOF software appear to ignore these variations, but it cannot be rigorously eliminated as a possible source of the variation we observed in our data. What is unequivocal is that the final data exhibits binning and, considering the bin width observed between 13 and 20 ppm, variation errors less than one bin width could have a significant impact.

While our initial data suggested to us variation in the flight time measurement is important in our observed data variability, further evaluation suggests calibration may be more critical. After collection of all laser shots, the data are combined and calibrated through a calibration function based on known internal masses (5-point nonlinear internal calibration in our experiments). The discontinuous binning of the flight time measurements would impact the peak interpolation algorithms for peptide masses and for calibration simultaneously. In these experiments, we are averaging data from multiple spectra of 500 laser shots/spectrum with 5-point internal calibration and a unique calibration for each spectrum (averaging 10 unique spectra with 10 unique calibration functions). This is fundamentally different than collecting individual spectra averaging 10,000 or more laser shots prior to calibration, which would provide the same number of combined laser shots, but with a single calibration function.

To evaluate these factors, we performed two experiments. First, we collected data at bin widths of 0.5, 1.0, 2.0 and 4.0 nanoseconds for 4 different peptides with 10 replicate measurements (S2 A—C Fig.). We anticipated that the standard deviation as a measure of variability would increase with increased bin width if binning were a factor contributing to variability. Our data shows that standard deviation is little impacted by increased bin width from 0.5 to 1.0 nanoseconds, but increased from 1.0 to 2.0 nanoseconds (S2 D Fig.). This data suggests a significant tolerance by the software interpolation algorithms for the number of data points across peaks when there are enough data points to define the peak. However, by 2.0 nanosecond bin width, the limits of the interpolation software are reached. For this reason, bin widths for data above 2.0 nanoseconds were unable to be calibrated. In the second experiment, we attempted to perform multiple calibrations on a single spectrum to determine if the calibration algorithm itself was contributing to the variances observed in the spectral data. However, recalibrating a single spectrum multiple times derived the identical calibration function and mass measurement, indicating that the calibration algorithm itself is not contributing to the observed variability. Initially, we thought this indicated that calibration was not important. However, calibration may be important when considering subtle differences present in multiple independent spectra. Therefore, we anticipate the observed variability in data is attributable to both the unique spectrum and the unique calibration function based on that spectrum. However, we also note that definitively determining the sources of the variation we observe in our MALDI-TOF data are beyond the capabilities of our instrumentation and resources.

### Spectral averaging improves accuracy and performance of MALDI-TOF mass spectrometry in protein identification

The Gaussian distribution of the observed data variability suggested that averaging multiple individual spectra would result in enhanced MALDI-TOF performance. The assignment of the MALDI-TOF data into bins places a significant limitation on the accuracy of the instrument. However, the electronic variability in the AD detector and calibration system can be exploited to provide random repositioning of the data bin positions by combining multiple independently calibrated acquisitions. This explains why our statistical mapping of the population of data for standard protein digest peptides provided more consistent mass measurements than individual spectra.

We tested this approach by identifying a protein by peptide mass fingerprinting from an immunoprecipitation experiment for β-Tubulin. β-Tubulin is an abundant cytoskeletal protein important in multiple cellular functions including cell structure, morphology, and intracellular trafficking[21]. Using a rabbit polyclonal antibody to this protein immobilized on Protein G Dynabeads, we immunoisolated β-Tubulin from HEK293 cell lysate and confirmed using Western blot (S3 Fig.). The remaining immunoisolated β-Tubulin was reduced and alkylated prior to digestion with trypsin and analysis by MALDI-TOF mass spectrometry. As described for the standard protein digest, 20 mass spectra were obtained for this digest, between 17 and 20 individual mass measurements were observed for each peptide, and the data were subjected to descriptive statistical analysis as described for data from the standard protein digestion (Table 1). The β-Tubulin digest was also analyzed using high-resolution LC-MS/MS for confirmation of mass measurement accuracy obtained on the MALDI-TOF instrument. For the majority of peptides analyzed, these data clearly demonstrate the same advantages from the spectrum averaging approach as described above for the standard protein digests. These peptide mass data was employed for peptide mass fingerprinting using ProFound[17] and successfully identified β-Tubulin from the immunoprecipitation sample using mass data from all observed peptides, even those peptides identified from LC-MS/MS as not derived from tubulin (Table 2). We note that identification of a protein from accurate mass measurements where peptides from multiple proteins are represented is inherently problematic for peptide mass fingerprinting. As expected, the identification of tubulin became more tenuous with increased stringency on accuracy. However, employing some of the tools suggested by the spectrum averaging experiments for the standard digests provided enhanced identification of tubulin. The standard peptide experiments demonstrated a standard deviation between 0.010–0.014 amu. In the Tubulin IP experiment, 5 of the 12 peptides identified, exhibit deviations above this range. Two of these peptides were identified by LC-MS/MS as derived from tubulin. Four deviations are minor, but with restricting the peptides for a second round of analysis to those identified to tubulin in the initial fingerprinting analysis and removing the two peptides with high standard deviations, the identification of tubulin was enhanced. Such an approach improves the confidence of a correct identification, but further experiments using alternative techniques are necessary for confirmation.

**Table 2:** Identification of tubulin peptides by ProFound peptide mass fingerprinting using immunoprecipitation experiments and averaged MALDI-TOF/TOF data.

Accuracy	Top Protein ID	Expectation	Peptide Set	Search Mass/pI Range
50ppm	TUBB	2.2x10-3	All	0-3000kDa/0-14
25ppm	TUBB2A	0.011	All	0-3000kDa/0-14
10ppm	Tubulin Beta Proteins	0.074	All	0-3000kDa/0-14
50ppm	TUBB2A	2.7x10-4	Tubulin	0-3000kDa/0-14
25ppm	TUBB2A	1.4x10-3	Tubulin	0-3000kDa/0-14
10ppm	Tubulin Beta Proteins	7.8x10-3	Tubulin	0-3000kDa/0-14
5ppm	Tubulin Beta Proteins	0.011–0.021	Tubulin	0-3000kDa/0-14

Tubulin was immunoprecipitated from HEK293 cell lysate using a rabbit polyclonal antibody and Protein A/G Dynabeads. After isolation, all eluted proteins were reduced and alkylated, digested with trypsin, and analyzed on an ABI 4800 MALDI-TOF/TOF mass spectrometer. Multiple individual spectra were acquired with internal calibration and between 10 and 23 individual measurements for each peptide were used for calculating the average observed masses for each peptide. Mass Tolerance is in parts-per-million (ppm), Peptide Set defines the peptides included for search and Search Mass Range/pI Range are input parameters for Profound. Top Protein ID and Expectation Value were calculated within ProFound from the mass spectrometry data using the IPI Human database (2010-02-01).

While protein identification using ProFound was effective for the tubulin IP, there was an unexpected error (Table 3). The peptide identified as ALTVSELTQQMFDSK (MH+ = 1697.8415) in ProFound was alternatively identified using LC-MS/MS sequence fragmentation data as NSSYFVEWIPNNVK (MH+ = 1696.8329) with an N-terminal asparagine deamidation to produce the sequence DSSYVEWIPNNVK (MH+ = 1697.817) (Table 3). The observed ion mass in the MALDI-TOF instrument was 1697.813, which is an error of −2.4 ppm with the peptide ion sequence assigned by LC-MS/MS analysis, but 17 ppm error for the assigned sequence from ProFound ([Ion Mass Observed—Ion Mass Theoretical] / Ion Mass Theoretical). In addition to the issues noted for peptide mass fingerprinting with peptides derived from multiple proteins, these data are important reminders of the limitations for protein identification based on accurate mass measurements alone.

**Table 3:** Beta-Tubulin identification and limitations of peptide mass fingerprinting.

Mass Observed	Mass Expected	Error (PPM)	Residue	Peptide Sequence
1129.581	1129.588	−6	242–251	FPGQLNADLR
1142.624	1142.627	−2	253–262	LAVNMVPFPR
1445.735	1445.681	37	325–336	EVDEQMLNVQNK
1619.824	1619.828	−2	263–276	LHFFMPGFAPLTSR
*1696.805	*1696.833	*-17	*283–297	*ALTVSELTQQMFDSK

The identification of tubulin from peptide mass fingerprinting matches with LC-MS/MS data for the majority of peptides. However, assignment for the 1249.585 and 1696.805 peptides were inaccurate. The 1249.585 peak was not assigned in the MALDI-TOF data. The observed mass of 1696.805 and data in Table 3 are from ProFound and assigned to peptide sequence ALTVSELTQQMFDSK (data denoted with an asterisk *). Peptide fragmentation data using LC-MS/MS suggests this assignment is incorrect and that the correct sequence is NSSYFVEWIPNNVK with deamidation at the amino-terminus yielding the sequence DSSYFVEWIPNNVK (MH+ = 1697.817 expected, 1697.813 observed, −2.4 ppm error).

We note that several immunoprecipitation peptide data standard deviations were not grossly out of proportion compared to test peptides, yet 3 peptides exhibited errors > 10 ppm (YMACCLLYR line 15, EVDEQMLNVQNK line 17, and LISWYDNEFGYSNR line 22, Table 1). Analysis of the raw data for these measurements demonstrated a systematic error resulting in the shifting of all measurements in the same direction. Unfortunately, systematic errors in a mass spectrometer are difficult to identify without external mass information and statistical analysis cannot resolve this problem. However, we were able to identify these errors effectively using the LC-MS/MS identifications. For one specific peptide, THNLEPYFESFINNLR (Line 23 and 24, Table 1), the magnitude of standard deviation was an effective indicator of measurement errors. Using all mass measurement data for this peptide (N = 19), we observed a Standard Deviation of 0.298 ppm and a calculated error of −52.268 ppm (Line 24, Table 1). These values are vastly greater than expected from standard peptide data. The Grubbs outlier test indicated two individual measurements were outside of the Grubbs test limit. We note that the Grubbs test is only valid for a single outlier measurement and we did not apply the test recursively. Removal of these outlier measurements (N = 17) vastly improved the quality of the data for this peptide. (Standard Deviation = 0.015 amu, error = −2.649 ppm, Line 23, Table 1).

The averaging approach used in this study was tested up to 2465.1983 amu (ACTH 18–39 Human) because of our confirmation of correct identification of peptides by MS/MS. The tuning and calibration for our 4800 MALDI-TOF/TOF system is optimized for this lower mass range because of high performance in the lower masses and problematic and unreliable peptide fragmentation above approximately 3500–4000 amu. However, the reflectron system on this instrument is capable of higher mass analyses for mass measurements of parent masses, but not for fragment masses. We tested the higher mass reflectron system on our instrument using Insulin (Monoisotopic Mass = 5803.6376, Average Mass = 5807.57) with the acknowledged limitations that the reflectron system was not tuned for this higher mass. As a consequence, the observed data has lower sensitivity, the resolution is unable to define monoisotopic peaks, and observed masses are consistent with average mass rather than monoisotopic mass. Despite these issues, we observed exactly the same type of variability as observed in the lower mass range with a majority of single mass observations inferior to the average of the population of mass observations (S3 Table). We fully expect that proper tuning and calibration of the reflectron system of the 4800 MALDI-TOF/TOF for higher masses such as insulin would provide similar advantages as observed in the properly tuned and calibrated lower mass region. However, the capabilities of this instrument to provide high accuracy reflectron data across a wide mass range from <1000 to >5000 amu will require further experiments. These data demonstrate that, given that similar analog-to-digital detection systems are utilized in all current MALDI-TOF instruments and that the same detection systems are used in both reflectron and linear modes, the averaging methods described in this article should be applicable across all mass ranges and modes. Obviously, reflectron data will be limited to lower mass range than linear data, but exhibit much higher accuracy as long as the reflectron system is tuned and calibrated for the appropriate mass ranges under study. Analog-to-digital detection systems are not limited to MALDI-TOF instruments, but are also incorporated into LC-TOF and LC-Q-TOF instruments as well. We expect that the same averaging approach used to enhance the mass accuracy and consistency in the AB Sciex 4800 MALDI-TOF/TOF mass spectrometer can be applied to mass spectrometers of LC-TOF and LC-Q-TOF design.

These studies did not utilize peak intensity as a parameter for averaging. Peak intensity can provide valuable data for quantitation and more consistent and reproducible quantitation would be highly advantageous. Additionally, peak intensity averaging could be readily incorporated into automated software platforms. Careful analysis of the raw peptide intensity data demonstrate that the variability in intensity measurements are much more pronounced than variability in mass measurements in the MALDI-TOF instrument (S1 and S2 Tables). This does not imply that averaging would not improve the consistency of intensity measurements and quantitation capabilities of this instrument, but that the larger standard deviations observed would be potentially problematic for comparing multiple data sets. We note that the peak intensities observed in the LC-Q-TOF instrument were far more consistent and reproducible. However, given the high performance of current LC-TOF type instruments such as the Bruker MicroTOF-Q instrument used in these studies, the gain in data quality by averaging would be expected to be much smaller than observed for MALDI-TOF instruments.

## Conclusions

In this article, we demonstrate a simple technique to improve MALDI-TOF instrument performance using spectral averaging. While LC-MS approaches have become predominant in recent biological research, enhanced performance MALDI-TOF instruments have unique and important capabilities for multiple biological fields including biochemistry, cell and molecular biology, chemistry, protein sciences, natural products, and microbial research. Highlighting the importance of these instruments is the recent FDA approval of a pathogen identification system based on MALDI-TOF mass spectrometry platform (FDA 501(k) Approval Letter)[22]. The theoretical limits for MALDI-TOF mass spectrometers far exceed any currently available instrument and we anticipate the future development of instruments with enhanced performance characteristics[20]. The spectrum averaging and descriptive statistics analysis described here can be easily incorporated into automated data collection algorithms and enhance the consistency and data quality for both current and future generation MALDI-TOF instruments.

## Supporting Information

S1 Fig A schematic representation of a modern reflector-based MALDI-TOF Mass Spectrometer. (DOCX)

S2 Fig Overlay of 10 individual spectra for monoisotopic peak at 2045 at bin size 0.5 nanoseconds (A), 1.0 nanoseconds (B), 2.0 nanoseconds (C), and Bin width and standard deviation correlation (D). (DOCX)

S3 Fig Immunoaffinity isolation of β-tubulin. (DOCX)

S1 Table Raw data for standard peptide measurements. (DOCX)

S2 Table Raw data for tubulin immunoprecipitation peptide measurements. (DOCX)

S3 Table Raw data for higher mass range insulin data. (DOCX)
