# Supplementary material for: Enhancing MALDI Time-Of-Flight Mass Spectrometer Performance through Spectrum Averaging
Source: PLoS One. 2015 Mar 23;10(3):e0120932. doi: 10.1371/journal.pone.0120932 (PMC4370844; doi:10.1371/journal.pone.0120932)
Supplement: S1 Fig — (DOCX) [file pone.0120932.s001.docx]

**
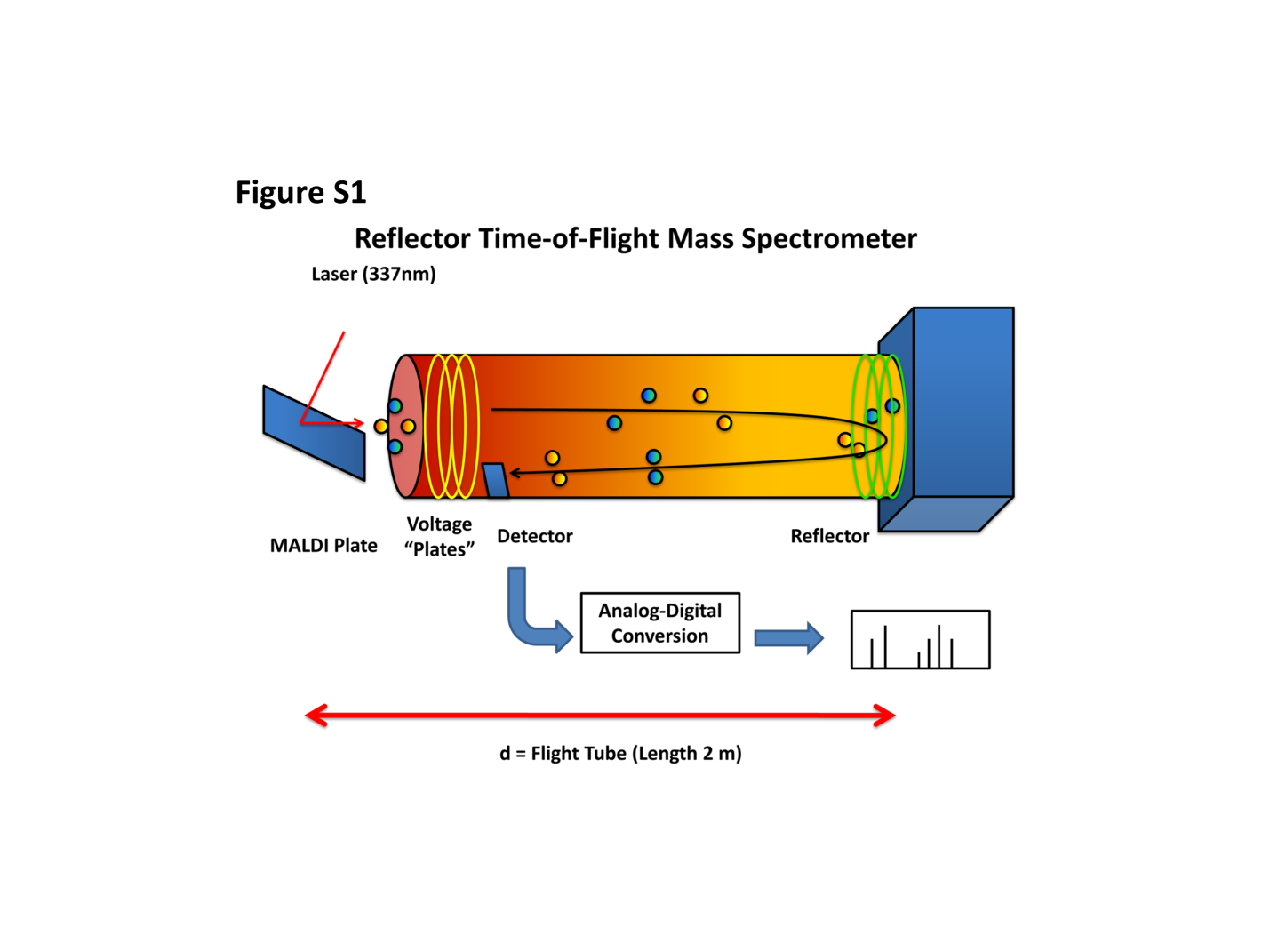
 S1 Fig. A schematic representation of a modern reflector-based MALDI-TOF mass spectrometer.** Modern high resolution MALDI-TOF mass spectrometers accelerate molecular analyte ions after laser ablation from a matrix and analyte mixture dried on a MALDI plate (dried-droplet method). After acceleration, the ions move through a field-free region until they are refocused and redirected by the reflector system. The ions time-of-flight are measured from the time of laser pulse to impact at the detector. These time measurements are recorded in the time domain through the analog-to-digital conversion (AD) detection system and converted to a mass spectrum.

**S1 Fig.**
