# Supplementary material for: Enhancing MALDI Time-Of-Flight Mass Spectrometer Performance through Spectrum Averaging
Source: PLoS One. 2015 Mar 23;10(3):e0120932. doi: 10.1371/journal.pone.0120932 (PMC4370844; doi:10.1371/journal.pone.0120932)
Supplement: S2 Fig — (DOCX) [file pone.0120932.s002.docx]

**
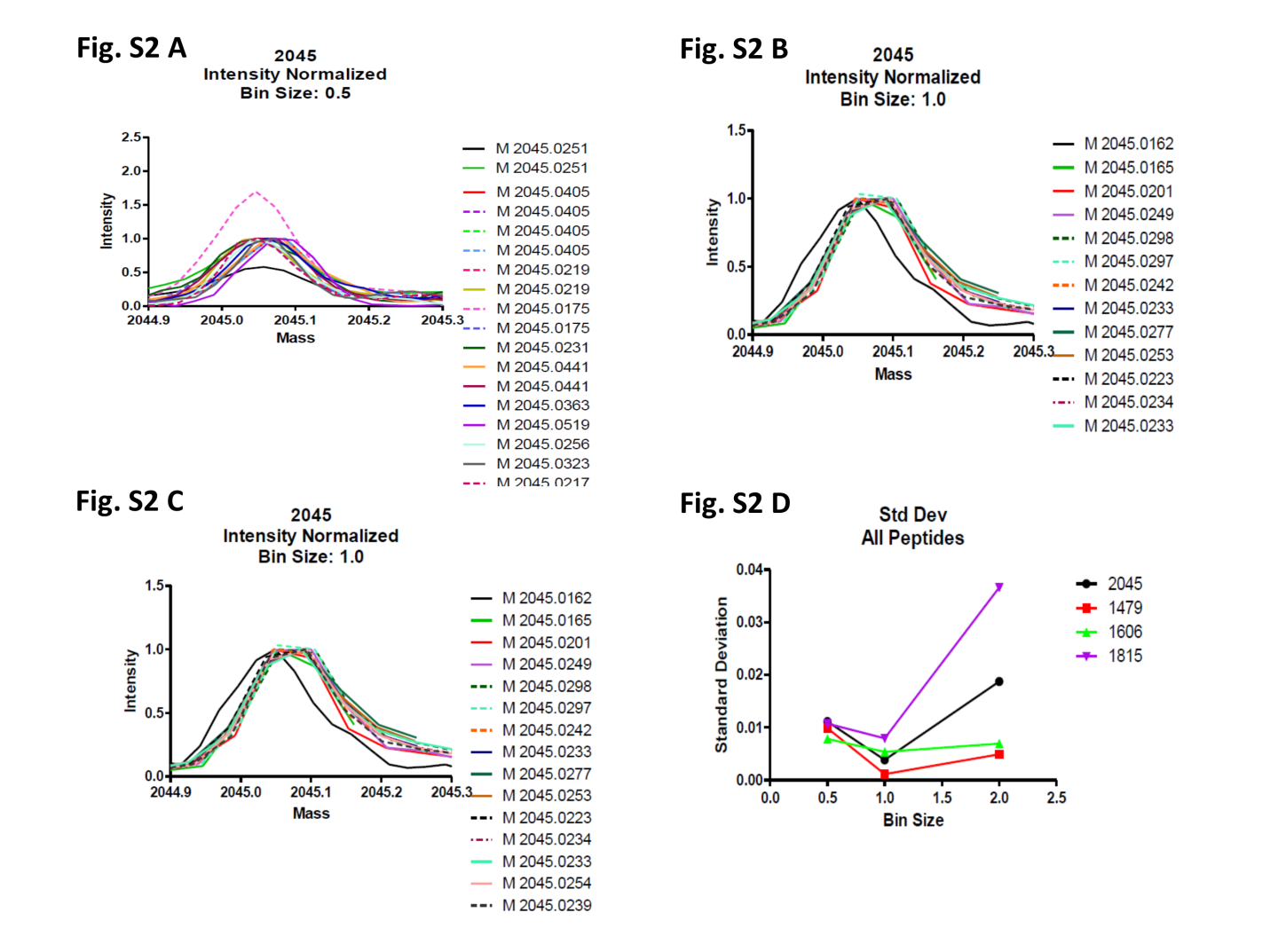
S2 Fig. Overlay of 10 individual spectra for monoisotopic peak at 2045 at bin size 0.5 nanoseconds (A), 1.0 nanoseconds (B), 2.0 nanoseconds (C) and bin width and standard deviation correlation (D).** Intensity was normalized to 1.0 for each spectral peak. Increasing bin width to 1.0 nanoseconds initially improves standard deviation, but extending to wider bin widths to 2.0 nanoseconds provides fewer sampling points across the peak leading to increased data variability. This variability is quantitated by standard deviation for the data.

**S2 D Fig.**

**S2 C Fig.**

**S2 A Fig.**

**S2 B Fig.**
