# Supplementary material for: Enhancing MALDI Time-Of-Flight Mass Spectrometer Performance through Spectrum Averaging
Source: PLoS One. 2015 Mar 23;10(3):e0120932. doi: 10.1371/journal.pone.0120932 (PMC4370844; doi:10.1371/journal.pone.0120932)
Supplement: S3 Fig — (DOCX) [file pone.0120932.s003.docx]

**
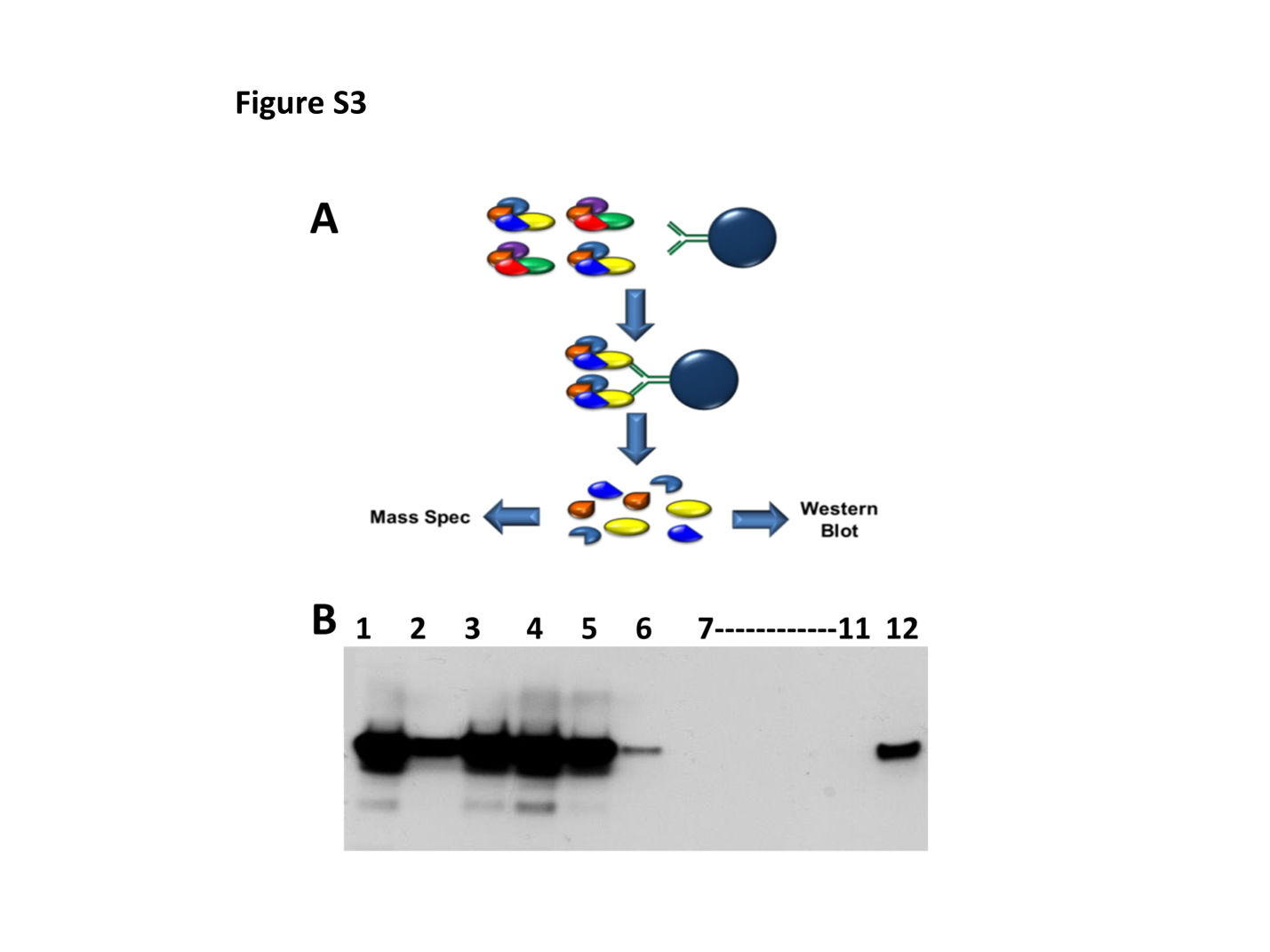
** **S3 Fig. Immunoaffinity isolation of β-tubulin.** (**A**) Schematic for immunoaffinity isolation. The antibody is immobilized on a solid resin support and the specific protein interacting with this antibody is isolated. Under appropriate conditions, proteins interacting with the specific target protein can also be isolated (co-immunoisolation or coimmunoprecipitation). (**B**) Western blot for immunoaffinity isolation of β-tubulin. Lane 1 = Cell Lysate, Lane 2 = Unbound Supernatent, Lane 3-11 = Wash Steps (various Tween 20 and NaCl concentrations), Lane 11 = Acid Elution, Lane 12 = Base Elution.

**S3 Fig.**
