# Supplementary material for: Enhancing MALDI Time-Of-Flight Mass Spectrometer Performance through Spectrum Averaging
Source: PLoS One. 2015 Mar 23;10(3):e0120932. doi: 10.1371/journal.pone.0120932 (PMC4370844; doi:10.1371/journal.pone.0120932)
Supplement: S1 Table — (DOCX) [file pone.0120932.s004.docx]

**S1 Table. Raw data for Standard Peptide Measurements.** Raw mass spectrometry measurements for Observed Mass (Obs. Mass), Intensity, Signal-to-Noise (S/N), Difference between calculated and measured masses (Δ or Delta) in amu and ppm are reported. Observed mass, Signal-to-Noise, and Intensity measurements are derived from the AB Sciex 4000 Series Data Explorer Software used for data acquisition and processing. The Δ mass differences were calculated using the standard equation:

**Δ = (Mass Observed – Mass Calculated)/Mass Calculated**

Where Mass Calculated is the accurate monoisotopic mass calculated from the primary sequence of the observed peptide. This Δ was multiplied by 10^6^ to convert to parts-per-million (ppm).

**S1 TABLE. RAW DATA FOR STANDARD PEPTIDE MEASUREMENTS**

RHPYFYAPELLYYANK

Obs. Mass Intensity S/N Δ (amu) Δ (ppm)

2045.0316 633.7260 561.0000 0.0036 1.7604

2045.0383 1118.2000 732.0000 0.0103 5.0366

2045.0219 390.9020 453.0000 -0.0061 -2.9828

2045.0319 639.4900 672.0000 0.0039 1.9071

2044.9935 2036.0800 1102.0000 -0.0345 -16.8702

2045.0161 1871.6900 1061.0000 -0.0119 -5.8190

2045.0286 960.6280 682.0000 0.0006 0.2934

2045.0248 281.7250 236.0000 -0.0032 -1.5648

2045.0099 811.2940 522.0000 -0.0181 -8.8507

2045.0137 553.4120 462.0000 -0.0143 -6.9926

2045.0248 1295.0600 615.0000 -0.0032 -1.5648

2045.0089 2138.3500 1084.0000 -0.0191 -9.3397

2045.0294 1817.1000 1073.0000 0.0014 0.6846

2044.9933 2977.8800 1373.0000 -0.0347 -16.9680

2045.0239 1626.3500 1180.0000 -0.0041 -2.0049

2045.0148 2976.0000 1347.0000 -0.0132 -6.4547

LGEYGFQNALIVR

Obs. Mass Intensity S/N Δ (amu) Δ (ppm)

1479.7771 616.7840 322.0000 -0.0183 -12.3666

1479.8064 1926.9000 585.0000 0.0110 7.4335

1479.7968 70.2745 33.0000 0.0014 0.9461

1479.7969 217.0980 145.0000 0.0015 1.0137

1479.7762 2511.0600 773.0000 -0.0192 -12.9748

1479.7689 3049.4100 1064.0000 -0.0265 -17.9079

1479.7799 532.0780 323.0000 -0.0155 -10.4744

1479.7697 764.8630 533.0000 -0.0257 -17.3673

1479.7953 1262.4300 486.0000 -0.0001 -0.0676

1479.7944 1064.1600 457.0000 -0.0010 -0.6758

1479.7815 1686.5900 461.0000 -0.0139 -9.3932

1479.7906 555.2940 157.0000 -0.0048 -3.2437

1479.7919 688.3140 328.0000 -0.0035 -2.3652

1479.8104 507.6080 125.0000 0.0150 10.1365

1479.8003 2870.5900 749.0000 0.0049 3.3113

1479.8043 2751.3700 736.0000 0.0089 6.0143

DAFLGSFLYEYSR

Obs. Mass Intensity S/N Δ (amu) Δ (ppm)

1567.7467 3195.6100 1698.0000 0.0040 2.5514

1567.7439 3013.0200 2029.0000 0.0012 0.7654

1567.7460 6049.8800 2539.0000 0.0033 2.1049

1567.7473 4340.0800 1865.0000 0.0046 2.9342

1567.7498 12876.5000 4012.0000 0.0071 4.5288

1567.7433 6143.3700 2600.0000 0.0006 0.3827

1567.7288 14824.8000 3303.0000 -0.0139 -8.8663

1567.7440 13323.9000 4164.0000 0.0013 0.8292

1567.7418 11107.1000 2983.0000 -0.0009 -0.5741

1567.7306 9724.2400 3854.0000 -0.0121 -7.7181

1567.7367 614.2750 456.0000 -0.0060 -3.8272

1567.7588 1517.1800 742.0000 0.0161 10.2695

1567.7484 564.7060 408.0000 0.0057 3.6358

1567.6989 203.2940 156.0000 -0.0438 -27.9383

1567.7388 3209.4100 1556.0000 -0.0039 -2.4877

1567.7219 428.5490 265.0000 -0.0208 - 13.2675

KVPQVSTPTLVEVSR

Obs. Mass Intensity S/N Δ (amu) Δ (ppm)

1639.9293 1487.0600 542.0000 -0.0084 -5.1221

1639.9491 1701.0200 455.0000 0.0114 6.9515

1639.9393 198.9020 87.0000 0.0016 0.9756

1639.9106 1242.3500 372.0000 -0.0271 -16.5250

1639.9260 371.4510 203.0000 -0.0117 -7.1344

1639.9492 181.9610 92.0000 0.0115 7.0125

1639.9453 404.0780 212.0000 0.0076 4.6343

1639.9283 1813.3300 410.0000 -0.0094 -5.7319

1639.9274 997.6470 344.0000 -0.0103 -6.2807

1639.9115 1619.4500 577.0000 -0.0262 -15.9762

1639.9308 1723.6100 664.0000 -0.0069 -4.2075

1639.9518 1286.9000 441.0000 0.0141 8.5979

1639.9446 1273.7300 418.0000 0.0069 4.2075

1639.9255 2705.5700 273.0000 -0.0122 -7.4393

1639.9404 2628.3900 627.0000 0.0027 1.6464

VPQVSTPTLVEVSR

Obs. Mass Intensity S/N Δ (amu) Δ (ppm)

1511.8123 93.4090 13.0000 -0.0305 -20.1741

1511.8214 114.1960 19.0000 -0.0214 -14.1549

1511.8217 75.9216 16.0000 -0.0211 -13.9565

1511.8177 213.9610 39.0000 -0.0251 -16.6023

1511.8218 72.7843 23.0000 -0.0210 -13.8903

1511.8285 225.8820 40.0000 -0.0143 -9.4587

1511.8440 133.0200 26.0000 0.0012 0.7937

1511.8293 80.3137 25.0000 -0.0135 -8.9295

1511.8754 65.8824 22.0000 0.0326 21.5631

1511.8131 62.7451 17.0000 -0.0297 -19.6449

1511.7979 77.1765 24.0000 -0.0449 -29.6989

1511.8529 300.5490 40.0000 0.0101 6.6806

1511.8536 175.6860 16.0000 0.0108 7.1436

1511.8566 253.4900 21.0000 0.0138 9.1279

1511.7921 115.4510 13.0000 -0.0507 -33.5352

1511.8289 128.6270 16.0000 -0.0139 -9.1941

GLSDGEWQQVLNVWGK

Obs. Mass Intensity S/N Δ (amu) Δ (ppm)

1815.9091 1092.3900 463.0000 0.0067 3.6896

1815.9031 399.0590 208.0000 0.0007 0.3855

1815.9071 1638.2700 659.0000 0.0047 2.5882

1815.9006 2598.2700 728.0000 -0.0018 -0.9912

1815.8884 2239.3700 590.0000 -0.0140 -7.7097

1815.8798 862.7450 245.0000 -0.0226 -12.4456

1815.9034 1567.3700 734.0000 0.0010 0.5507

1815.8903 3737.1000 881.0000 -0.0121 -6.6634

1815.8629 249.0980 119.0000 -0.0395 -21.7523

1815.8971 154.9800 71.0000 -0.0053 -2.9187

1815.9027 140.5490 64.0000 0.0003 0.1652

1815.8977 1240.4700 305.0000 -0.0047 -2.5882

1815.9015 1169.5700 294.0000 -0.0009 -0.4956

1815.8918 2152.1600 584.0000 -0.0106 -5.8373

1815.8987 373.3330 137.0000 -0.0037 -2.0376

1815.9082 410.9800 176.0000 0.0058 3.1940

VEADIAGHGQEVLIR

Obs. Mass Intensity S/N Δ (amu) Δ (ppm)

1606.8483 7198.7500 2599.0000 -0.0064 -3.9829

1606.8442 8803.7600 2697.0000 -0.0105 -6.5345

1606.8591 1118.1200 633.0000 0.0044 2.7383

1606.8544 4875.2900 1163.0000 -0.0003 -0.1867

1606.8459 2202.9800 1212.0000 -0.0088 -5.4765

1606.8688 1528.4700 687.0000 0.0141 8.7749

1606.8646 2230.5900 1118.0000 0.0099 6.1611

1606.8480 7589.0200 1589.0000 -0.0067 -4.1696

1606.8469 4859.6100 1627.0000 -0.0078 -4.8542

1606.8550 8126.7500 2234.0000 0.0003 0.1867

1606.8508 5423.0600 1808.0000 -0.0039 -2.4271

1606.8712 6115.1400 2035.0000 0.0165 10.2685

1606.8643 5677.1800 1889.0000 0.0096 5.9744

1606.8452 9697.2500 2165.0000 -0.0095 -5.9122

1606.8599 11731.5000 2746.0000 0.0052 3.2361

1606.8307 14322.8000 2693.0000 -0.0240 -14.9360

1606.8580 5672.1600 1515.0000 0.0033 2.0537

1606.8309 10701.8000 2007.0000 -0.0238 -14.8115

1606.8342 14566.3000 2479.0000 -0.0205 -12.7578

1606.8390 10336.6000 2264.0000 -0.0157 -9.7706

1606.8527 15350.6000 2693.0000 -0.0020 -1.2447

1606.8511 17577.4000 3169.0000 -0.0036 -2.2404

1606.8519 18396.2000 2793.0000 -0.0028 -1.7425

KGHHEAELKPLAQSHATK

Obs. Mass Intensity S/N Δ (amu) Δ (ppm)

1982.0525 6893.1800 2254.0000 -0.0041 -2.0686

1982.0385 8739.1400 2719.0000 -0.0181 -9.1319

1982.0703 7203.7700 1959.0000 0.0137 6.9120

1982.0646 4702.7500 1784.0000 0.0080 4.0362

1982.0452 9069.1800 3053.0000 -0.0114 -5.7516

1982.0422 2302.1200 1173.0000 -0.0144 -7.2652

1982.0432 1356.5500 903.0000 -0.0134 -6.7607

1982.0653 2581.3300 972.0000 0.0087 4.3894

1982.0607 10199.8000 2609.0000 0.0041 2.0686

1982.0608 11536.9000 2395.0000 0.0042 2.1190

1982.0583 7072.6300 2287.0000 0.0017 0.8577

1982.0564 5056.6300 1773.0000 -0.0002 -0.1009

1982.0587 3272.7800 1676.0000 0.0021 1.0595

1982.0338 7292.2400 2261.0000 -0.0228 -11.5032

1982.0533 3527.5300 1628.0000 -0.0033 -1.6649

1982.0146 1180.8600 571.0000 -0.0420 -21.1901

1982.0697 1595.6100 1257.0000 0.0131 6.6093

1982.0602 1921.8800 2311.0000 0.0036 1.8163

1982.0535 5150.1200 1963.0000 -0.0031 -1.5640

GHHEAELKPLAQSHATK

Obs. Mass Intensity S/N Δ (amu) Δ (ppm)

1853.9457 307.4510 83.0000 -0.0160 -8.6302

1853.9475 542.7450 129.0000 -0.0142 -7.6593

1853.9702 490.6670 110.0000 0.0085 4.5848

1853.9718 353.2550 84.0000 0.0101 5.4478

1853.9487 868.6470 220.0000 -0.0130 -7.0120

1853.9603 1500.2400 311.0000 -0.0014 -0.7551

1853.9509 681.4120 156.0000 -0.0108 -5.8254

1853.9520 841.4120 198.0000 -0.0097 -5.2320

1853.9475 739.1370 194.0000 -0.0142 -7.6593

1853.9502 860.2350 255.0000 -0.0115 -6.2029

1853.9714 581.6470 144.0000 0.0097 5.2320

1853.9841 666.3530 181.0000 0.0224 12.0822

1853.9543 385.8820 202.0000 -0.0074 -3.9915

1853.9581 143.0590 71.0000 -0.0036 -1.9418

1853.9528 573.4900 224.0000 -0.0089 -4.8005

1853.9331 65.2549 29.0000 -0.0286 -15.4264

1853.9512 304.9410 153.0000 -0.0105 -5.6635

1853.9529 305.5690 143.0000 -0.0088 -4.7466

1853.9731 98.5098 74.0000 0.0114 6.1490

1853.9624 304.3140 136.0000 0.0007 0.3776

HKIPIKYLEFISDAIIHVLHSK

Obs. Mass Intensity S/N Δ (amu) Δ (ppm)

2601.4846 559.6860 369.0000 -0.0069 -2.6523

2601.4795 731.6080 523.0000 -0.0120 -4.6127

2601.4983 355.7650 259.0000 0.0068 2.6139

2601.4563 183.8430 182.0000 -0.0352 -13.5307

2601.4900 317.4900 256.0000 -0.0015 -0.5766

2601.4800 607.3730 404.0000 -0.0115 -4.4205

2601.4700 220.8630 183.0000 -0.0215 -8.2645

2601.5078 267.9220 215.0000 0.0163 6.2656

2601.4880 101.0200 107.0000 -0.0035 -1.3454

2601.4756 129.8820 102.0000 -0.0159 -6.1119

2601.4844 149.3330 75.0000 -0.0071 -2.7292

2601.4836 116.7060 75.0000 -0.0079 -3.0367

YLEFISDAIIHVLHSK

Obs. Mass Intensity S/N Δ (amu) Δ (ppm)

1884.9935 868.1370 331.0000 -0.0283 -15.0131

1885.0079 619.9220 338.0000 -0.0139 -7.3739

1884.9883 1184.6300 558.0000 -0.0335 -17.7717

1885.0328 1035.9200 342.0000 0.0110 5.8355

1885.0220 1812.7100 605.0000 0.0002 0.1061

1885.0074 767.3730 356.0000 -0.0144 -7.6392

1885.0027 766.7450 300.0000 -0.0191 -10.1325

1885.0236 915.4510 276.0000 0.0018 0.9549

1885.0151 759.2160 413.0000 -0.0067 -3.5543

1885.0131 1985.2600 612.0000 -0.0087 -4.6153

1885.0260 1153.2500 389.0000 0.0042 2.2281

1885.0145 904.7840 346.0000 -0.0073 -3.8726

1885.0149 415.3730 254.0000 -0.0069 -3.6604

1885.0264 276.0780 197.0000 0.0046 2.4403

1885.0245 2362.3500 731.0000 0.0027 1.4323
