# Supplementary material for: Enhancing MALDI Time-Of-Flight Mass Spectrometer Performance through Spectrum Averaging
Source: PLoS One. 2015 Mar 23;10(3):e0120932. doi: 10.1371/journal.pone.0120932 (PMC4370844; doi:10.1371/journal.pone.0120932)
Supplement: S2 Table — (DOCX) [file pone.0120932.s005.docx]

**S2 Table. Raw data for Tubulin Immunoprecipitation Measurements.** Raw mass spectrometry measurements for Observed Mass (Obs. Mass), Intensity, Signal-to-Noise (S/N), Difference between calculated and measured masses (Δ or Delta) in amu and ppm are reported. Observed mass, Signal-to-Noise, and Intensity measurements are derived from the AB Sciex 4000 Series Data Explorer Software used for data acquisition and processing. The Δ mass differences were calculated using the standard equation:

**Δ = (Mass Observed – Mass Calculated)/Mass Calculated**

Where Mass Calculated is the accurate monoisotopic mass calculated from the primary sequence of the observed peptide. This Δ was multiplied by 10^6^ to convert to parts-per-million (ppm).

**S2 TABLE. RAW DATA FOR TUBULIN IMMUNOPRECIPITATION PEPTIDES MEASUREMENTS**

LHFFMPGFAPLTSR

Obs. Mass Intensity S/N Δ (amu) Δ (ppm)

1620.8340 284.2000 158.0000 -0.0015 -0.9254

1620.8313 2264.8000 352.0000 -0.0042 -2.5913

1620.8467 2919.5000 410.0000 0.0112 6.9100

1620.8317 1777.9000 334.0000 -0.0038 -2.3445

1620.8323 2614.0000 243.0000 -0.0032 -1.9743

1620.8304 3937.9000 439.0000 -0.0051 -3.1465

1620.8296 4183.8000 422.0000 -0.0059 -3.6401

1620.8313 193.6000 91.0000 -0.0042 -2.5913

1620.8256 1514.4000 210.0000 -0.0099 -6.1080

1620.8157 4015.7000 251.0000 -0.0198 -12.2159

1620.8254 2653.8000 184.0000 -0.0101 -6.2314

1620.8328 1742.7000 191.0000 -0.0027 -1.6658

1620.8293 2443.0000 361.0000 -0.0062 -3.8252

1620.8411 2960.6000 284.0000 0.0056 3.4550

1620.8306 1425.6000 263.0000 -0.0049 -3.0231

1620.8367 897.9000 148.0000 0.0012 0.7404

1620.8486 252.9000 159.0000 0.0131 8.0823

1620.8259 2801.3000 208.0000 -0.0096 -5.9229

1620.8229 3149.8000 370.0000 -0.0126 -7.7738

1620.8313 2264.8000 352.0000 -0.0042 -2.5913

NSSYFVEWIPNNVK

Obs. Mass Intensity S/N Δ (amu) Δ (ppm)

1697.8187 1124.1000 64.0000 0.0017 1.0488

1697.8158 2264.8000 352.0000 -0.0012 -0.7404

1697.8286 285.2000 53.0000 0.0116 7.1568

1697.8098 1777.9000 334.0000 -0.0072 -4.4422

1697.7910 2614.0000 40.0000 -0.0260 -16.0411

1697.8146 3937.9000 92.0000 -0.0024 -1.4807

1697.8000 4183.8000 67.0000 -0.0170 -10.4884

1697.7859 552.5000 39.0000 -0.0311 -19.1876

1697.7955 338.1000 38.0000 -0.0215 -13.2648

1697.8129 1742.7000 55.0000 -0.0041 -2.5296

1697.8208 2443.0000 68.0000 0.0038 2.3445

1697.8361 2960.6000 50.0000 0.0191 11.7840

1697.8069 1452.6000 52.0000 -0.0101 -6.2314

1697.8322 72.8000 45.0000 0.0152 9.3779

1697.8206 88.5000 27.0000 0.0036 2.2211

1697.8198 2801.3000 50.0000 0.0028 1.7275

1697.8063 3149.8000 83.0000 -0.0107 -6.6015

1697.8158 2264.8000 57.0000 -0.0012 -0.7404

THNLEPYFESFINNLR

Obs. Mass Intensity S/N Δ (amu) Δ (ppm)

1993.9747 175.1000 87.0000 -0.0020 -1.0030

1993.9679 2264.8000 125.0000 -0.0088 -4.4133

1993.0447 408.2000 13.0000 -0.9320 -467.4077

1993.9845 1777.9000 141.0000 0.0078 3.9118

1993.0112 168.5000 11.0000 -0.9655 -484.2083

1993.9785 3937.9000 155.0000 0.0018 0.9027

1993.9716 4183.8000 114.0000 -0.0051 -2.5577

1993.9602 1514.4000 121.0000 -0.0165 -8.2749

1993.9445 4015.7000 87.0000 -0.0322 -16.1486

1993.9512 2653.8000 78.0000 -0.0255 -12.7885

1993.9691 1742.7000 158.0000 -0.0076 -3.8115

1993.9966 2443.0000 103.0000 0.0199 9.9801

1993.9904 2960.6000 103.0000 0.0137 6.8707

1993.9758 1425.6000 95.0000 -0.0009 -0.4514

1993.9452 897.9000 83.0000 -0.0315 -15.7976

1993.9885 119.2000 77.0000 0.0118 5.9178

1993.9866 2801.3000 107.0000 0.0099 4.9650

1993.9680 3149.8000 97.0000 -0.0087 -4.3631

1993.9679 2264.8000 125.0000 -0.0088 -4.4133

FPGQLNADLR

Obs. Mass Intensity S/N Δ (amu) Δ (ppm)

1130.5896 1124.1000 27.0000 -0.0057 -5.0416

1130.5834 2264.8000 46.0000 -0.0119 -10.5254

1130.5975 2919.5000 37.0000 0.0022 1.9459

1130.5815 1777.9000 29.0000 -0.0138 -12.2060

1130.5901 2614.0000 58.0000 -0.0052 -4.5993

1130.5942 3937.9000 80.0000 -0.0011 -0.9729

1130.5771 217.7000 40.0000 -0.0182 -16.0977

1130.6061 1451.9000 20.0000 0.0108 9.5525

1130.5895 1514.4000 48.0000 -0.0058 -5.1300

1130.5825 300.2000 52.0000 -0.0128 -11.3215

1130.5759 147.5000 34.0000 -0.0194 -17.1591

1130.5850 1742.7000 55.0000 -0.0103 -9.1102

1130.5957 2443.0000 60.0000 0.0004 0.3538

1130.5927 2960.6000 53.0000 -0.0026 -2.2997

1130.5837 1425.6000 39.0000 -0.0116 -10.2601

1130.5830 897.9000 32.0000 -0.0123 -10.8792

1130.5911 608.9000 26.0000 -0.0042 -3.7149

1130.6007 2801.3000 54.0000 0.0054 4.7762

1130.5895 3149.8000 31.0000 -0.0058 -5.1300

1130.5834 2264.8000 46.0000 -0.0119 -10.5254

SYELPDGQVITIGNER

Obs. Mass Intensity S/N Δ (amu) Δ (ppm)

1790.8918 257.6000 125.0000 -0.0001 -0.0558

1790.8939 2264.8000 180.0000 0.0020 1.1168

1790.9143 2919.5000 198.0000 0.0224 12.5077

1790.9121 1777.9000 85.0000 0.0202 11.2793

1790.8721 318.4000 20.0000 -0.0198 -11.0559

1790.8917 3937.9000 195.0000 -0.0002 -0.1117

1790.8698 4183.8000 119.0000 -0.0221 -12.3402

1790.8884 1451.9000 47.0000 -0.0035 -1.9543

1790.8827 1514.4000 60.0000 -0.0092 -5.1371

1790.8754 82.2000 13.0000 -0.0165 -9.2133

1790.9044 54.0000 21.0000 0.0125 6.9798

1790.8903 1742.7000 83.0000 -0.0016 -0.8934

1790.9014 2443.0000 99.0000 0.0095 5.3046

1790.9117 2960.6000 70.0000 0.0198 11.0559

1790.8899 1425.6000 160.0000 -0.0020 -1.1168

1790.9098 50.0000 24.0000 0.0179 9.9950

1790.9166 608.9000 51.0000 0.0247 13.7920

1790.8826 2801.3000 85.0000 -0.0093 -5.1929

1790.8900 3149.8000 70.0000 -0.0019 -1.0609

1790.8939 2264.8000 180.0000 0.0020 1.1168

AMGIMNSFVNDIFER

Obs. Mass Intensity S/N Δ (amu) Δ (ppm)

1743.8141 257.6000 124.0000 -0.0052 -2.9820

1743.8230 469.3000 184.0000 0.0037 2.1218

1743.8444 2919.5000 50.0000 0.0251 14.3937

1743.8269 1777.9000 201.0000 0.0076 4.3582

1743.8306 2614.0000 133.0000 0.0113 6.4800

1743.8282 3937.9000 282.0000 0.0089 5.1037

1743.8171 4183.8000 119.0000 -0.0022 -1.2616

1743.8329 1451.9000 89.0000 0.0136 7.7990

1743.8176 1514.4000 136.0000 -0.0017 -0.9749

1743.8077 4015.7000 202.0000 -0.0116 -6.6521

1743.8145 388.1000 171.0000 -0.0048 -2.7526

1743.8306 234.7000 114.0000 0.0113 6.4800

1743.8226 374.9000 185.0000 0.0033 1.8924

1743.8245 2960.6000 157.0000 0.0052 2.9820

1743.8328 323.8000 145.0000 0.0135 7.7416

1743.8201 106.4000 54.0000 0.0008 0.4588

1743.8373 608.9000 134.0000 0.0180 10.3222

1743.8184 2801.3000 147.0000 -0.0009 -0.5161

1743.8127 3149.8000 226.0000 -0.0066 -3.7848

1743.8230 469.3000 184.0000 0.0037 2.1218

LDIDSPPITAR

Obs. Mass Intensity S/N Δ (amu) Δ (ppm)

1197.6628 1124.1000 25.0000 0.0154 12.8585

1197.6691 1150.7000 16.0000 0.0217 18.1189

1197.6493 91.9000 10.0000 0.0019 1.5864

1197.6340 101.3000 17.0000 -0.0134 -11.1886

1197.6147 1054.7000 17.0000 -0.0327 -27.3035

1197.6462 117.0000 21.0000 -0.0012 -1.0020

1197.6238 120.5000 17.0000 -0.0236 -19.7053

1197.6605 184.8000 20.0000 0.0131 10.9381

1197.6449 121.1000 25.0000 -0.0025 -2.0874

1197.6674 77.8000 21.0000 0.0200 16.6994

1197.6746 144.6000 23.0000 0.0272 22.7112

1197.6658 85.3000 26.0000 0.0184 15.3635

1197.6600 872.2000 14.0000 0.0126 10.5206

1197.6526 51.8000 10.0000 0.0052 4.3418

1197.6760 82.2000 21.0000 0.0286 23.8802

1197.6552 98.5000 27.0000 0.0078 6.5128

1197.6691 86.9000 16.0000 0.0217 18.1189

YMACCLLYR

Obs. Mass Intensity S/N Δ (amu) Δ (ppm)

1249.5958 342.9000 11.0000 0.0432 34.5724

1249.5830 2264.9000 38.0000 0.0304 24.3287

1249.5831 2919.5000 28.0000 0.0305 24.4087

1249.5880 53.3000 11.0000 0.0354 28.3301

1249.6208 1054.7000 21.0000 0.0682 54.5795

1249.6110 2144.0000 32.0000 0.0584 46.7367

1249.5707 4183.8000 1268.0000 0.0181 14.4852

1249.5692 559.1000 12.0000 0.0166 13.2848

1249.5697 4015.7000 52.0000 0.0171 13.6849

1249.5630 92.5000 25.0000 0.0104 8.3230

1249.5732 2443.0000 31.0000 0.0206 16.4859

1249.5985 2960.6000 806.0000 0.0459 36.7331

1249.5839 872.2000 14.0000 0.0313 25.0490

1249.5646 507.0000 14.0000 0.0120 9.6034

1249.5725 51.8000 14.0000 0.0199 15.9257

1249.6105 71.5000 17.0000 0.0579 46.3366

1249.5815 3149.8000 28.0000 0.0289 23.1283

1249.5830 2264.8000 38.0000 0.0304 24.3287

IWHHTFYNELR

Obs. Mass Intensity S/N Δ (amu) Δ (ppm)

1515.7432 1124.1000 47.0000 -0.0060 -3.9584

1515.7533 2919.5000 154.0000 0.0041 2.7049

1515.7452 1777.9000 116.0000 -0.0040 -2.6390

1515.7428 152.8000 60.0000 -0.0064 -4.2223

1515.7532 3937.9000 145.0000 0.0040 2.6390

1515.7435 4183.8000 147.0000 -0.0057 -3.7605

1515.7462 1451.9000 39.0000 -0.0030 -1.9792

1515.7361 4015.7000 140.0000 -0.0131 -8.6426

1515.7445 2653.8000 99.0000 -0.0047 -3.1008

1515.7551 1742.7000 68.0000 0.0059 3.8925

1515.7509 2443.0000 92.0000 0.0017 1.1216

1515.7516 2960.6000 99.0000 0.0024 1.5834

1515.7538 1425.6000 62.0000 0.0046 3.0348

1515.7361 897.9000 67.0000 -0.0131 -8.6426

1515.7531 608.9000 103.0000 0.0039 2.5730

1515.7537 2801.3000 62.0000 0.0045 2.9688

1515.7430 3149.8000 124.0000 -0.0062 -4.0904

1515.7386 194.5000 91.0000 -0.0106 -6.9932

LISWYDNEFGYSNR

Obs. Mass Intensity S/N Δ (amu) Δ (ppm)

1763.8040 1124.1000 146.0000 0.0016 0.9071

1763.8088 469.3000 76.0000 0.0064 3.6285

1763.8434 743.8000 79.0000 0.0410 23.2452

1763.8241 104.2000 37.0000 0.0217 12.3030

1763.8174 318.4000 51.0000 0.0150 8.5044

1763.8192 894.7000 63.0000 0.0168 9.5249

1763.8257 867.8000 68.0000 0.0233 13.2101

1763.8412 162.2000 24.0000 0.0388 21.9979

1763.7992 205.2000 41.0000 -0.0032 -1.8143

1763.8038 552.5000 36.0000 0.0014 0.7937

1763.8323 388.1000 33.0000 0.0299 16.9520

1763.8137 234.7000 51.0000 0.0113 6.4066

1763.8173 374.9000 56.0000 0.0149 8.4477

1763.8236 110.4000 43.0000 0.0212 12.0195

1763.8483 323.8000 34.0000 0.0459 26.0233

1763.8477 106.4000 21.0000 0.0453 25.6831

1763.8396 174.1000 16.0000 0.0372 21.0908

1763.7982 320.0000 45.0000 -0.0042 -2.3812

1763.8212 578.8000 44.0000 0.0188 10.6588

1763.8088 469.3000 76.0000 0.0064 3.6285

LAVNMVPFPR

Obs. Mass Intensity S/N Δ (amu) Δ (ppm)

1143.6235 56.2000 13.0000 -0.0108 -9.4436

1143.6239 1150.7000 24.0000 -0.0104 -9.0938

1143.6345 152.2000 34.0000 0.0002 0.1749

1143.6284 101.3000 11.0000 -0.0059 -5.1590

1143.6174 180.7000 23.0000 -0.0169 -14.7775

1143.6324 294.9000 38.0000 -0.0019 -1.6614

1143.6300 4183.8000 51.0000 -0.0043 -3.7599

1143.6365 108.2000 11.0000 0.0022 1.9237

1143.6458 107.6000 12.0000 0.0115 10.0557

1143.6287 4015.7000 78.0000 -0.0056 -4.8967

1143.6259 2653.8000 53.0000 -0.0084 -7.3450

1143.6370 167.2000 21.0000 0.0027 2.3609

1143.6268 73.7000 24.0000 -0.0075 -6.5580

1143.6399 166.6000 36.0000 0.0056 4.8967

1143.6313 97.3000 21.0000 -0.0030 -2.6232

1143.6796 50.0000 14.0000 0.0453 39.6106

1143.6145 50.0000 13.0000 -0.0198 -17.3132

1143.6366 178.5000 24.0000 0.0023 2.0111

1143.6267 130.5000 42.0000 -0.0076 -6.6455

1143.6239 70.0000 24.0000 -0.0104 -9.0938

EVDEQMLNVQNK

Obs. Mass Intensity S/N Δ (amu) Δ (ppm)

1446.7217 811.6000 469.0000 0.0324 22.3960

1446.7548 53.0000 13.0000 0.0655 45.2758

1446.7471 218.4000 23.0000 0.0578 39.9533

1446.7390 152.8000 16.0000 0.0497 34.3543

1446.7378 372.1000 29.0000 0.0485 33.5248

1446.7341 471.5000 24.0000 0.0448 30.9673

1446.7483 54.9000 12.0000 0.0590 40.7828

1446.7224 140.5000 23.0000 0.0331 22.8798

1446.7360 280.2000 19.0000 0.0467 32.2806

1446.7550 202.0000 20.0000 0.0657 45.4140

1446.7312 52.4000 15.0000 0.0419 28.9627

1446.7456 50.0000 14.0000 0.0563 38.9164

1446.7909 56.5000 20.0000 0.1016 70.2293

1446.7365 50.0000 17.0000 0.0472 32.6262

1446.7352 192.9000 17.0000 0.0459 31.7276

1446.7433 204.2000 26.0000 0.0540 37.3266

1446.7548 53.0000 13.0000 0.0655 45.2758
