# Supplementary material for: Enhancing MALDI Time-Of-Flight Mass Spectrometer Performance through Spectrum Averaging
Source: PLoS One. 2015 Mar 23;10(3):e0120932. doi: 10.1371/journal.pone.0120932 (PMC4370844; doi:10.1371/journal.pone.0120932)
Supplement: S3 Table — (DOCX) [file pone.0120932.s006.docx]

**S3 Table. Raw Data for Higher Mass Range Insulin Data.** To confirm the observed variability at lower mass range also occurs at higher mass range using the reflectron system in the 4800 MALDI-TOF/TOF mass spectrometer, we added insulin (Monoisotopic Mass = 5803.6376, Average Mass = 5807.57) to our standard peptide mixture for internal calibration and the data for 30 replicate measurements for insulin were acquired and internally calibrated as described above. All data reported and calculated in this Table use the same methods as described for **Tables S1** and **S2**. The 4800 mass spectrometer was not tuned for this higher mass region, which resulted in lower intensity and resolution of the observed insulin mass signal. The observed masses were consistent with the average mass of insulin because of the lower intensity and resolution. Despite these limitations, the identical variability observed in the lower mass regions was reproduced in this higher mass region. As reported for the lower mass region, the average of the population of data provided a significantly more accurate measurement of the insulin mass than the majority of single measurements (18/30 single measurements were less accurate than average of all measurements). We anticipate optimization of the higher mass region of the 4800 MALDI-TOF/TOF mass spectrometer reflectron system will enhance the accuracy of the insulin measurement.

**S3 TABLE. RAW DATA FOR HIGHER MASS RANGE INSULIN DATA**

Obs. Mass S/N Δ (amu) Δ (ppm)

5808.31 49 0.73 125.697795

5809.20 38 1.62 278.9457915

5809.70 31 2.12 365.0401716

5809.73 23 2.15 370.2058344

5807.85 37 0.27 46.49096526

5809.90 13 2.32 399.4779237

5808.14 23 0.56 96.42570572

5807.44 14 -0.14 -24.10642643

5806.59 29 -0.99 -170.4668726

5809.73 13 2.15 370.2058344

5806.92 36 -0.66 -113.6445817

5807.48 19 -0.1 -17.21887602

5805.71 11 -1.87 -321.9929816

5807.73 22 0.15 25.82831403

5804.94 13 -2.64 -454.5783269

5807.74 19 0.16 27.55020163

5809.86 17 2.28 392.5903733

5807.26 13 -0.32 -55.10040327

5811.25 24 3.67 631.93275

5808.18 24 0.6 103.3132561

5808.00 55 0.42 72.31927929

5807.98 10 0.4 68.87550408

5809.13 13 1.55 266.8925783

5810.21 15 2.63 452.8564393

5808.42 21 0.84 144.6385586

5806.70 15 -0.88 -151.526109

5810.10 17 2.52 433.9156757

5808.55 22 0.97 167.0230974

5809.54 10 1.96 337.48997

5808.68 24 1.1 189.4076362

Insulin Monoisotopic Mass = 5803.638 amu

Insulin Average Mass = 5807.58 amu

Data Average Mass = 5808.366 amu

Data Average Delta (ppm) = 135.283
